# Water Deficit-Responsive QTLs for Cell Wall Degradability and Composition in Maize at Silage Stage

**DOI:** 10.3389/fpls.2019.00488

**Published:** 2019-04-25

**Authors:** Laëtitia Virlouvet, Fadi El Hage, Yves Griveau, Marie-Pierre Jacquemot, Emilie Gineau, Aurélie Baldy, Sylvain Legay, Christine Horlow, Valérie Combes, Cyril Bauland, Carine Palafre, Matthieu Falque, Laurence Moreau, Sylvie Coursol, Valérie Méchin, Matthieu Reymond

**Affiliations:** ^1^Institut Jean-Pierre Bourgin, INRA, AgroParisTech, CNRS, Université Paris-Saclay, Versailles, France; ^2^Univ. Paris-Sud, Université Paris-Saclay, Orsay, France; ^3^Génétique Quantitative et Evolution - Le Moulon, INRA, Université Paris-Sud, CNRS, AgroParisTech, Université Paris-Saclay, Gif-sur-Yvette, France; ^4^Unité Expérimentale du Maïs, INRA, Saint-Martin-de-Hinx, France

**Keywords:** cell wall composition, cell wall degradability, drought response, quantitative trait locus, constitutive QTL, responsive QTL, maize

## Abstract

The use of lignocellulosic biomass for animal feed or biorefinery requires the optimization of its degradability. Moreover, biomass crops need to be better adapted to the changing climate and in particular to periods of drought. Although the negative impact of water deficit on biomass yield has often been mentioned, its impact on biomass quality has only been recently reported in a few species. In the present study, we combined the mapping power of a maize recombinant inbred line population with robust near infrared spectroscopy predictive equations to track the response to water deficit of traits associated with biomass quality. The population was cultivated under two contrasted water regimes over 3 consecutive years in the south of France and harvested at silage stage. We showed that cell wall degradability and β-O-4-linked H lignin subunits were increased in response to water deficit, while lignin and *p*-coumaric acid contents were reduced. A mixed linear model was fitted to map quantitative trait loci (QTLs) for agronomical and cell wall-related traits. These QTLs were categorized as “constitutive” (QTL with an effect whatever the irrigation condition) or “responsive” (QTL involved in the response to water deficit) QTLs. Fifteen clusters of QTLs encompassed more than two third of the 213 constitutive QTLs and 13 clusters encompassed more than 60% of the 149 responsive QTLs. Interestingly, we showed that only half of the responsive QTLs co-localized with constitutive and yield QTLs, suggesting that specific genetic factors support biomass quality response to water deficit. Overall, our results demonstrate that water deficit favors cell wall degradability and that breeding of varieties that reconcile improved drought-tolerance and biomass degradability is possible.

## Introduction

Besides its use as animal feed, lignocellulosic biomass is increasingly used for the production of second generation biofuel or chemical building blocks (Torres et al., 2015; Vermerris and Abril, 2015; Bichot et al., 2018). Many of these uses require an efficient enzymatic degradation of the biomass. Degradability is measured as the percentage of lignocellulosic biomass that is assimilated by an animal or converted into sugars in a biorefinery process. A major limiting factor in these processes is the accessibility of structural polysaccharides to the hydrolytic enzymes, which is frequently prevented by the phenolic polymer fraction. In this context, a major challenge for plant breeding is to limit biomass recalcitrance without affecting crop yield, pest resistance, or drought tolerance (van der Weijde et al., 2013).

Lignocellulosic biomass consists primarily of cell wall polymers. Studies (reported in Vogel, 2008) have shown that grass cell walls are composed of cellulose, hemicelluloses, and phenolics approximately in a 45-45-10 ratio. These values vary between developmental stages, plant tissues and genotypes. Cell wall degradability was shown to be strongly influenced by the content of phenolic compounds such as lignin and *p*-hydroxycinnamic acids (Hartley, 1972; Grabber et al., 1998; Casler and Jung, 1999; Méchin et al., 2001; Jung and Casler, 2006; Zhang et al., 2011; El Hage et al., 2018). In addition, the nature of the linkages between lignin subunits also plays an important role as shown for instance by the negative correlation between the amount of ß-O-4-linkages in the lignin polymers and cell wall degradability among samples with comparable lignin contents (Zhang et al., 2011). The influence of the proportion of guiacyl (G), syringil (S), and *p*-hydroxyphenyl (H) lignin subunits on cell wall degradability is still controversial, and conclusions differ depending one studies lignified cell walls (Grabber et al., 2003; Jung and Casler, 2006; de Oliveira et al., 2015) in dicotyledons (Baucher et al., 1999; Casler and Jung, 1999; Goujon et al., 2003), or in grasses (Méchin et al., 2000; Zhang et al., 2011;El Hage et al., 2018).

Grasses are particularly rich in *p*-hydroxycinnamic acids, among which *p*-coumaric acids are found mainly esterified (PCAest) to S lignin units (Ralph et al., 1994; Grabber et al., 1996; Lu and Ralph, 1999) and ferulic acids (FA) associated with hemicelluloses and/or lignins through ester (FAest) or ether (FAeth) linkages, respectively (Hatfield et al., 2017). In maize, PCAest content was shown to be negatively correlated with cell wall degradability (Gabrielsen et al., 1990; Méchin et al., 2000; Taboada et al., 2010; Zhang et al., 2011; El Hage et al., 2018) and FA content might also affect cell wall degradability (Barrière et al., 2005).

Biosynthesis and regulatory pathways for cell wall components have been intensively studied (Boerjan et al., 2003; Vanholme et al., 2008; Riboulet et al., 2009; Gray et al., 2012) and numerous genes encoding critical enzymes and transcription factors have been identified. In addition, over the last two decades, over 400 quantitative trait loci (QTLs) underlying natural variation in cell wall composition and degradability have been identified in maize using mainly bi-parental populations (Lübberstedt et al., 1997b,c; Bohn et al., 2000; Barrière et al., 2001, 2008, 2012; Méchin et al., 2001; Roussel et al., 2002; Cardinal et al., 2003; Fontaine et al., 2003; Papst et al., 2004; Cardinal and Lee, 2005; Krakowsky et al., 2005, 2006; Riboulet et al., 2008a; Wei et al., 2009; Lorenzana et al., 2010; Torres et al., 2015; Leng et al., 2018). A QTL meta-analysis identified 26 meta-QTLs for cell wall degradability and 42 meta-QTLs for cell wall (Truntzler et al., 2010), suggesting a complex genetic determinism, which however, might be reduced to a smaller number of genomic regions. Interestingly, only few major QTLs (which explained more than 20% of the observed variation) for cell wall-related traits have been found (Roussel et al., 2002; Courtial et al., 2013) and only less than half of all the meta-QTLs for cell wall degradability co-localized with meta-QTLs for cell wall composition, underscoring the fact that cell wall composition and degradability have complex genetic determinisms. Despite the fact that yield and degradability are often negatively correlated (Barrière et al., 2004), breeding forage maize hybrids for increased cell wall degradability, without impacting yield turned out to be possible (Baldy et al., 2017).

In the context of global climate change, many scenarios predict more frequent drought periods, which, together with dwindling fresh water supplies, are expected to have strong impacts on crop yields (Samaniego et al., 2018; Webber et al., 2018). Water deficit affects, within minutes, physiological processes underlying leaf and root growth, such as cell division, hydraulics, cell wall mechanics, and primary and secondary metabolism., This is likely to have long lasting consequences (days to months) on whole-plant transpiration and water uptake and as a result, on biomass yield and quality (for reviews see Reynolds and Langridge, 2016; Tardieu et al., 2018). In addition, crop plants under water deficit often contain excess carbon and roots and reproductive organs frequently appear to experience sink limitation. Furthermore, under agronomical conditions, deregulation of the synchronization of male and female flowering time are often reported under water deficit, leading to grain abortion and massive yield loss (Denmead and Shaw, 1960; Reynolds and Langridge, 2016; Turc and Tardieu, 2018). The genetic determinism of drought tolerance in maize grain yield has been extensively studied (Ribaut and Ragot, 2007; Collins et al., 2008; Ribaut et al., 2010) and interestingly, recent reports showed that water deficit positively impacts cell wall degradability in maize (Emerson et al., 2014; El Hage et al., 2018), sorghum (Perrier et al., 2017), miscanthus (Emerson et al., 2014; van der Weijde et al., 2017), and sugarcane (dos Santos et al., 2015). However, so far, no QTL for cell wall degradability or composition in response to water deficit have been reported.

In the present study, we performed QTL mapping using a maize recombinant inbred line (RIL) population derived from a cross between two parental inbred lines, F271 and Cm484, to explore the genetic factors underlying variation of cell wall-related traits in response to water deficit in maize plant. This population was cultivated in field trials over three consecutive years under both irrigated and non-irrigated scenarios. Firstly, we determined the impact of a non-irrigated scenario on cell wall-related traits using maize stover and dedicated near infrared spectroscopy (NIRS) predictive equations. Secondly, analyzing jointly the data from both irrigation scenarios, we were able to demonstrate that allelic variation (F271 vs. Cm484) at several loci were responsible for observed phenotypic variation whatever the irrigation scenario (constitutive QTLs, Collins et al., 2008). In addition, we also pointed out loci where allelic variation impacted the variation of quantified traits in response to irrigation scenario (responsive QTLs).

## Materials and Methods

### Plant Materials and Field Experiments

A RIL population consisting of 267 lines was developed at INRA by single seed descent (SSD) for 6 generations from a cross between maize inbred early lines F271 (INRA line bred from Canadian dent; Barrière et al., 2001; Roussel et al., 2002) and Cm484 (Canada-Morden−1989; Méchin et al., 2000; Barrière et al., 2007). All the RILs were planted in a randomized augmented bloc design with one replicate of both parents in each bloc over 3 years (2013, 2014 and 2015). Field trials were carried out in the South of Montpellier (France). This localization is under a Mediterranean climate characterized by warm and dry summers and humid winters. Over the last 30 years, the annual mean temperature and precipitation were 15.2°C and 629 mm, respectively. The mean summer temperature was 29.3°C (July) and over the last years, a tendency toward dryer summers was observed. The water deficit was estimated at 180 mm in June and July and around 100 mm in May and August (Delalande et al., 2017). The soil was of a stony loamy-clay nature, with a depth reaching up to 200 m. Plants were grown in open field under Irrigated (I) and Non-Irrigated (NI) conditions. Blocs of I and NI were adjacent in the same experimental fields each year and localized 15–20 m apart in order to prevent irrigation of the non-irrigated blocs. Fifteen repetitions in 2013 and eight repetitions in 2014 and 2015 were cultivated in each scenario. Under the I scenario, the water was supplied with a mobile ramp of sprinklers twice a week (30 mm of water supplied every time), and under the NI scenario water was no more supplied when the 5th leave of INRA check early line F2 showed the 5th leave ligulated until 14 days after all the RILs flowered. Each line was grown in a single 4.20 m row with 0.80 m between rows and a planting density of 80,000 plants/ha. At the silage stage, ears with husks and peduncle were removed manually from the plants just before the stover plots were machine-harvested with a forage chopper. In the field, plant height and biomass yield were determined. A representative sample of nearly 350 g fresh chopped stover per plot was collected for dry-matter (DM) content estimation and biochemical and NIRS analyses. All samples were dried in a forced-air oven at 55°C and ground with a hammer mill (1 mm grid).

### Establishment of Accurate NIRS Predictive Equations and Biochemical Analyses

Cell wall biochemistry-related traits of all the harvested dried samples were estimated using NIRS predictive equations. To do so, these equations were developed at INRA Versailles for maize plants without ears harvested at silage stage. Depending on the trait, 60–200 samples of maize stover from inbred lines harvested at silage stage were biochemically analyzed to calibrate and to validate the established equations. Out of these 200 samples, 49 were selected from the F271 × Cm484 RIL progeny evaluated in the I (23 samples) and NI (26 samples) scenarios over 3 years (6 and 9 samples from the I and NI scenarios, respectively, in 2013; 14 and 11 samples from the I and NI scenarios, respectively, in 2014; 3 and 6 samples from the I and NI scenarios, respectively, in 2015). This selection was carried out to make the predictive equations accurate for the samples harvested in the present study. Calibration equations were validated using a set of 20–40 calibration samples for all traits, except for neutral detergent fiber (NDF), acid detergent lignin (ADL.NDF), and polysaccharides (CL.NDF and HC.NDF), which were obtained by the Goering and Van Soest (1970) using a cross validation approach (Table 1).

**Table 1:** Characteristics of NIRS calibrations developed for cell wall traits in stover maize plants without ears.

**Categories**	**Traits^a^**	**Units**	**Range**	**Calibration**		**Validation**		
				***n***	***r***	***n***	***r***	**SECV**
Cell Wall residues	NDF	%DM	43.34–69.2	57	0.983	0	0.95	1.77
Degradability	IVDMD	%DM	32.3–66.9	160	0.963	38	0.93	2.22
	IVCWRD	%CWR	25.67–51.02	161	0.805	38	0.81	3.28
Lignin content	KL.CWR	%CWR	11.06–18.67	167	0.852	40	0.83	0.77
	ADL.NDF	%NDF	3.21–6.91	57	0.925	0	0.83	0.48
Lignin structure	bO4	μmole g^−1^ KL	255–1,023	168	0.893	40	0.90	86.00
	bO4.H	μmole g^−1^ KL	3–28.5	166	0.636	40	0.71	4.08
	bO4.G	μmole g^−1^ KL	132.15–564.24	166	0.869	40	0.88	50.80
	bO4.S	μmole g^−1^ KL	119.06–540.39	166	0.855	40	0.86	52.40
	S/G		0.46–1.68	166	0.709	40	0.62	0.19
*p*-Hydroxycinnamic acids	PCAest	mg g^−1^ CWR	4.66–17.94	164	0.864	39	0.78	1.71
	FAest	mg g^−1^ CWR	2.05–6.92	164	0.802	39	0.83	0.54
	Faeth	mg g^−1^ CWR	1.59–3.88	164	0.451	39	0.45	0.31
Structural sugars	CL.NDF	%NDF	43.42–54.48	57	0.903	0	0.78	1.52
	GLU	%CWR	28.73–43.35	81	0.835	19	0.76	3.22
	HC.NDF	%NDF	39.67–51.89	57	0.905	0	0.77	1.81
	ARA	%CWR	3–4.84	81	0.843	19	0.77	0.26
	GAL	%CWR	0.6–2.06	81	0.945	19	0.93	0.15
	XYL	%CWR	16.64–23.39	81	0.623	19	0.70	1.08

a *NDF, neutral detergent fiber; IVDMD, in vitro dry matter degradability; IVCWRD, in vitro cell wall residues degradability; ADL, acid detergent lignin; KL, Klason lignin; PCAest, esterified para-coumaric acid; FAeth, etherified ferulic acid; FAest, esterified ferulic acid; CL, cellulose; HC, hemicellulose; GLU, glucose; ARA, arabinose; GAL, galactose; XYL, xylose*.

Concerning the biochemical analyses performed on the calibration and validation sets, cell wall residue (CWR) was obtained with a water/ethanol extraction (Soxhlet). NDF, acid detergent fiber (ADF), ADL, cellulose [CL.NDF = 100^*^(ADF-ADL)/NDF] and hemicellulose [HC.NDF = 100^*^(NDF-ADF)/NDF] contents were estimated according to Goering and Van Soest (1970). Lignin content in the cell wall (KL.CWR) was estimated using the Klason method according to Dence (1992). Esterified and etherified *p*-hydroxycinnamic acids (PCAest, FAest, FAeth) contents were estimated after alkaline hydrolysis of the CWRs (Méchin et al., 2000; Culhaoglu et al., 2011). The monomeric structure and composition of lignin (units β-O-4.H, β-O-4.S, and β-O-4.G) was determined through the oxidation of CWRs by thioacidolysis (Lapierre et al., 1986). Glucose (GLU), Xylose (XYL), and Arabinose (ARA) contents were determined by acid hydrolysis (Updegraff, 1969; Harholt et al., 2006). The *in vitro* dry matter degradability (IVDMD) and cell wall residue degradability (IVCWRD) were estimated according to a modified protocol derived from Aufrère and Michalet-Doreau (1983). Briefly, 30 mg of dry matter was pretreated in an acid solution (HCL 0.1N) at 40°C for 24 h after which 2 M NaOH was added to terminate the reaction. The sample was then incubated in a cellulase solution (Cellulase Onozuka R10 8 mg.ml^−1^, NaAc 0.1M pH 4.95, Na_2_CO_3_ 0.4%) at 50°C during 72 h. After centrifugation, the pellet was washed with water and frozen before lyophilization and the weight loss was expressed as a percentage of the initial weight (30 mg).

### Statistical Analyses of the Cell Wall Dataset

All statistical analyses were performed using R software (R Core Team, 2014). To eliminate the environmental effects, single-plot values were corrected by a subtraction of the best linear unbiased prediction (BLUP) value of the bloc effect for each line, obtained using the following mixed linear model (1):

(1) $$\begin{array}{l} Y_{\text{ijkl}}=\text{μ}+g_{\text{i}}\left(1-{\text{t}}_{\text{i}}\right)+{C_{i}t}_{\text{i}}+y_{\text{j}}+{\text{e}}_{\text{k}}+{\text{B}}_{\text{jkl}}+{\text{g}}_{\text{i}}y_{\text{j}}\\ +g_{\text{i}}e_{\text{k}}+y_{\text{j}}e_{\text{k}}+{\text{E}}_{\text{ijkl}} \end{array}$$

where *Y*_ijkl_ is the phenotypic value of the *i*th line in the *j*th year, in the *k*th irrigation scenario and localized in the *l*th bloc in field. In this model, μ is the intercept. The genetic effect of line i is considered as fixed and noted *C*_*i*_ if *i* was one of the two parental lines used as checks and as random and noted *g*_i_ if *i* was one of the RILs. The parameter *t*_i_ was set to one for checks and zero otherwise. The genetic effects of the RILs were assumed to be independent and identically distributed. The B_jkl_ bloc effects were considered as random, as well as the interactions between the *g*_i_ genetic and the *y*_j_ year or the *e*_k_ irrigation scenario effects. The year *y*_j_ and irrigation scenario *e*_k_ effects as well as the interaction between them *ye*_jk_ were considered as fixed effects.

A principal component analysis (PCA) was carried out in order to reduce the number of traits to describe the variation of cell wall related traits over the corrected data set. The R package FactoMineR was used for the PCA (Lè et al., 2008).

Corrected data for each trait for the RILs were then used to estimate variance components and trait heritabilities, using the following model (2):

(2) $$\begin{array}{l} {Y^{\prime }}_{\text{ijk}}=\text{μ}+g_{\text{i}}+{gy_{i}}_{\text{j}}+{ge_{i}}_{\text{k}}+y_{\text{j}}+e_{\text{k}}+{ye_{j}}_{\text{k}}+{\text{E}}_{\text{ijk}} \end{array}$$

where *Y*′_ijk_ is the corrected phenotypic value of the “i”th line in the “j”th year, in the “k”th irrigation scenario. In this equation, the *g*_i_ represents the genetic effect, the *y*_j_ and the *e*_k_ the year and irrigation scenario effects, and the E_ijk_ the residual error effect.

Broad sense heritability was calculated using the variance components estimated with the linear model (2) where the *g*_i_ genetic effects and the interactions between the *g*_i_ genetic and the *y*_j_ year or the *e*_k_ irrigation scenario effects were considered as random effects, as follows:

$$\begin{array}{l} {\text{h}}^{2}=\sigma_{\text{g}}^{2}/\left[\sigma_{\text{g}}^{2}+\sigma_{\text{ge}}^{2}/\text{k}+\sigma_{\text{gy}}^{2}/\text{j}+\;\sigma_{\text{E}}^{2}/\text{(obs/i)}\right] \end{array}$$

where $\sigma_{\text{g}}^{2}$ represents the genetic variance, $\sigma_{\text{ge}}^{2}$ the variance of interaction between genotype and irrigation scenario, $\sigma_{\text{gy}}^{2}$ the variance of the interaction between genotype and year, $\sigma_{\text{E}}^{2}$ the residual error variance item, and k, j, obs, i the number of irrigation scenarios, year, observations and lines, respectively. The ratio “obs/i” therefore corresponds to the average number of observations per RIL over the whole experimental design.

The corrected data obtained with the model (1) were also used to compute least-square means (ls-means) of each recombinant inbred line using specific models. To obtain ls-means for lines using both irrigation conditions jointly (“all”), we used a linear model including genotype, year, irrigation scenario and the interaction between year and irrigation scenarios effects, all considered as fixed. For the ls-means of the irrigation conditions separately (“I” and “NI” for irrigated and non-irrigated, respectively), we used the linear model including only the genotype and year effects of the corrected data for irrigated or non-irrigated conditions, respectively.

### Genotyping and Genetic Map Construction

Leaf tissues were collected from all 261 RILs and parental inbred lines F271 and Cm484 and freeze-dried at −70°C. Genomic DNA was extracted using a procedure derived from Dellaporta et al. (1983), Michaels et al. (1994), and Tai and Tanksley (1990). Genomic DNA was used for genotyping using the genotyping by sequencing (GBS) approach (Elshire et al., 2011). Briefly, the genomic DNA was digested with the restriction enzyme ApeK1 and used to construct GBS libraries in 96-plex. The GBS libraries were sequenced by Illumina HiSeq2000 and SNP calling was performed using the TASSEL GBS pipeline with B73 as the reference genome (Glaubitz et al., 2014).

Initially, 955,720 markers were identified well-distributed on maize chromosomes 1 to 10. Among those, we selected 2,806 polymorphic markers between parental lines than 15% missing data among the RILs. These markers were then used to construct the linkage map using R scripts interacting with the CarthaGene software (de Givry et al., 2005), as described in Ganal et al. (2011). Specifically, a scaffold map of 1,775 cM containing 20 to 39 markers per chromosome was first built with very stringent criteria for order robustness (minimum spacing of 5cM between adjacent markers), and then densified with additional markers to produce a framework map containing 62 to 161 markers per chromosome while keeping a LOD threshold for order robustness greater or equal to 3.0. In total, 1,000 markers were retained following this procedure. The total length of the framework map was 2,355 cM with an average spacing of around 2.4 cM. By looking *a posteriori* at singletons in the dataset, we verified that the increase of the genetic map length when saturating the scaffold with additional markers was not attributable to genotyping errors, but more likely to the rather high level of missing data, which introduces a bias in the imputation procedure (EM algorithm) of CarthaGene as a result of genetic interference.

### QTL Detection

To integrate the response to water treatment, single-marker analysis was performed on the corrected data for each trait and the coordinates of the PCA components. The genome was scanned with the following mixed linear model (3):

(3) $$\begin{array}{l} {Y^{\prime }}_{\text{ijk}}=\text{μ}+g_{\text{i}}+y_{\text{j}}+e_{\text{k}}+y_{\text{j}}\text{x}{\text{e}}_{\text{k}}+m_{p}{\text{x}}_{\text{ip}}+m_{p}\text{x}{\text{e}}_{\text{kp}}x_{\text{ip}}+{\text{E}}_{\text{ijkp}} \end{array}$$

where *Y*′_ijkl_ is the corrected phenotypic value of the line *i* in the year *j*, in the irrigation scenario *k* and for the reference allele *p* at the tested marker. In this model, μ is the intercept and *g*_i._ the residual genetic effect, which not accounted for by the marker effect, was considered random. The 1,000 markers identified on the genetic map were analyzed one by one using their “p” allele genotype for all the lines. The markers with a *p*-value inferior to 0.005% were selected for the marker effect (named constitutive QTL) and for the interaction between the marker and the irrigation scenario (named responsive QTL). All the adjacent markers along the genetic map with a *p*-value inferior to 0.005% were considered to form a QTL and the confident interval of each QTL was then estimated by the distance of the most distant markers with a *p*-value inferior to 0.005%. To estimate the percentage of variance (*r*^2^) explained by each detected QTL, we used the linear model (4) including the irrigation scenario, the marker and the interaction between the marker and the irrigation scenario effects on the ls-mean data *per* irrigation scenario.

(4) $$\begin{array}{l} {Y^{\prime }}_{\text{ki}}=\text{μ}+e_{\text{k}}+m_{\text{p}}+m_{p}\text{x}{\text{e}}_{\text{k}}+{\text{E}}_{\text{ki}} \end{array}$$

where *Y*′_ik_ is the corrected phenotypic value of the line *i* in the irrigation scenario *k*.

To calculate the *r*^2^ explained by the QTL, we calculated the difference between *r*^2^ from the full model (4) and the *r*^2^ from the model (4) without the marker for the constitutive QTL or without the interaction between the marker and the irrigation scenario for the responsive QTL.

To calculate the effect of the QTL, we used the ls-mean data of both irrigation conditions jointly and separately (“I” and “NI” for irrigated and non-irrigated, respectively). For the constitutive QTL, the QTL effect was estimated at the marker position as the difference between the two parental allele effects divided by the range of variation of the trait on the recombinant inbred line population. For the responsive QTL, obtained with the “*m*_p_ x *e*_k_” factor, the QTL effect was calculated at the marker position by dividing the difference of the irrigation scenario response between the two parental allele with the range of variation of the response of the trait on the recombinant inbred progeny. Ls-mean data for every traits obtained for both irrigation conditions separately have been also used to detect QTL following standard MQM procedure in R proposed by Broman and Sen (2009).

## Results

### Two Parental Inbred Lines Have Contrasting Cell Wall Composition, Degradability, and Responses to Irrigation

To quantify 19 traits related to cell wall composition and degradability, we established predictive NIRS equations using maize stover samples harvested at silage stage over 3 years under both irrigation scenarios. The range of variation among the calibration samples was high for most of the traits and allowed for robust calibrations (Table 1) as shown by the high *r* values (ranging from 0.70 to 0.95) for all the traits except for S/G ratio (*r* = 0.62) and FAeth (*r* = 0.45). These equations therefore allowed a reliable prediction of the cell wall composition and structure of the samples from the RIL progeny.

Agronomic and cell wall-related traits were then evaluated for the two parental inbred lines F271 and Cm484 under the I scenario (Table 2). F271 produced more biomass than Cm484 and all 19 traits related to cell wall composition and degradability, except for FAest, were found distinct between the two parental inbred lines. F271 biomass was less degradable (IVDMD) and had a higher NDF and lignin content (LK.CWR and ADL.NDF) relative to Cm484. Lignin structure was also different between the two parental lines, F271 having more ß-O-4-linked lignin, higher PCAest and FAeth contents than Cm484. IVCWRD was also lower in F271 than in Cm484. Finally, the structural sugars GLU and XYL showed higher levels in F271 than Cm484, while HC.NDF, ARA and GAL levels were lower in F271 than in Cm484.

**Table 2:** Agronomical, cell wall composition, and degradability traits for the two parental inbred lines F271 and Cm484.

**Categories**	**Traits^e^**	**Units**	**Irrigated**		**Non-Irrigated**		**% of response**		**Genetic^g^**	**Irrigation scenario^g^**	**Genetic × Irrigation scenario^g^**	**Year^g^**	**Genetic × year^g^**	**Year × Irrigation scenario^g^**	**Residual^g^**
			**F271^f^**	**Cm484^f^**	**F271^f^**	**Cm484^f^**	**F271**	**Cm484**							
Agronomic	Plant height	cm	172.08^d^ ± 9.73	134.06^b^ ± 8.78	143.52^c^ ± 11.22	122.91^a^ ± 7.94	16.6	8.3	50.2^***^	24.3^***^	4.91^***^	0.17	9.59^***^	0.57	10.25
	Yield	t ha^−1^	3.83^c^ ± 0.36	2.48^b^ ± 0.39	2.43^ab^ ± 0.75	2.09^c^ ± 0.45	36.6	15.7	23.69^***^	29.43^***^	9.23^***^	2.16^*^	3.18^**^	1.22	31.06
Cell Wall residue	NDF	%DM	52.82^b^ ± 2.01	47.83^a^ ± 1.75	54.58^c^ ± 2.2	52.45^b^ ± 2.14	3.3	9.7	30.99^***^	24.02^***^	4.99^***^	2.65^*^	0.60	0.38	36.36
Degradability	IVDMD	%DM	46.78^a^ ± 2.38	55.68^c^ ± 1.88	50.95^b^ ± 2.39	56.28^c^ ± 2.35	8.9	1.1	63.2^***^	7.81^***^	4.13^***^	4.11^***^	0.04	1.11^*^	19.59
	IVCWRD	%CWR	30.11^a^ ± 2.25	36.49^b^ ± 1.42	38.16^c^ ± 2.43	43.19^d^ ± 2.31	26.7	18.4	30.27^***^	53.06^***^	0.47^*^	1.08^**^	0.76^*^	0.39	9.18
Lignin content	KL.CWR	%CWR	16.41^c^ ± 0.56	14.61^b^ ± 0.37	14.67^b^ ± 0.4	13.3^a^ ± 0.27	10.6	9.0	44.68^***^	42.49^***^	0.89^**^	0.49	0.79^*^	1.24^**^	9.38
	ADL.NDF	%NDF	5.74^c^ ± 0.36	4.75^b^ ± 0.21	4.66^b^ ± 0.26	3.85^a^ ± 0.18	18.8	18.9	38.42^***^	48.73^***^	0.44^*^	1.27^**^	1.05^**^	0.80^*^	9.28
Lignin structure	bO4	μmole g^−1^ KL	587.81^c^ ± 56.17	492.34^ab^ ± 51.9	529.41^b^ ± 64.09	475.02^a^ ± 71.58	9.9	3.5	24.94^***^	6.96^***^	2.07^*^	4.62^*^	2.28	3.48^*^	55.63
	bO4.H	μmole g^−1^ KL	15.24^b^ ± 1.27	13.94^a^ ± 0.98	17.8^c^ ± 1.75	17.18^c^ ± 1.15	16.6	23.2	5.82^***^	52.22^***^	0.68	3.63^**^	0.69	2.81^*^	34.13
	bO4.G	μmole g^−1^ KL	288.42^b^ ± 34.71	240.24^a^ ± 26.35	271.06^b^ ± 34.45	245.21^a^ ± 36.39	6.0	2.1	23.23^***^	0.87	2.34^*^	8.93^***^	2.31	2.89	59.41
	bO4.S	μmole g^−1^ KL	280.66^c^ ± 27.1	247.34^b^ ± 29.32	242.86^ab^ ± 30.01	224.33^a^ ± 38.35	13.5	9.3	11.88^***^	17.21^***^	1.08	5.03^*^	1.81	3.27	59.70
	S/G		0.95^b^ ± 0.08	1.00^c^ ± 0.05	0.86^a^ ± 0.06	0.90^a^ ± 0.06	9.5	10.0	7.35^***^	32.67^***^	0.25	17.05^***^	0.09	1.25	41.33
*p*-Hydroxycinnamic acids	PCAest	mg g^−1^ CWR	14.04^c^ ± 1.24	11.43^b^ ± 0.73	10.94^b^ ± 0.81	8.68^a^ ± 0.66	22.1	24.1	33.23^***^	49.45^***^	0.19	1.25^**^	0.49	1.56^**^	13.80
	FAest	mg g^−1^ CWR	3.52^b^ ± 0.34	3.51^b^ ± 0.39	3.24^a^ ± 0.34	3.38^ab^ ± 0.31	8.0	3.7	0.92	8.08^***^	1.21	3.22^*^	0.77	21.73^***^	64.04
	Faeth	mg g^−1^ CWR	2.57^b^ ± 0.11	2.50^a^ ± 0.13	2.50^a^ ± 0.08	2.45^a^ ± 0.07	2.7	2.0	7.58^***^	6.58^***^	0.51	22.81^***^	0.14	22.29^***^	40.06
Structural sugars	CL.NDF	%NDF	50.38^b^ ± 1.62	49.87^ab^ ± 1.2	50.24^b^ ± 1.25	49.2^a^ ± 0.93	0.3	1.3	8.47^***^	2.41.	1	6.88^*^	0.06	14.33^***^	66.84
	GLU	%CWR	39.67^c^ ± 1.23	38.01^b^ ± 1.04	37.53^b^ ± 0.85	36.43^a^ ± 0.99	5.4	4.2	19.32^***^	37.87^***^	0.86.	14.64^***^	0.18	2.65^**^	24.46
	HC.NDF	%NDF	43.84^a^ ± 1.97	45.33^b^ ± 1.36	45.1^b^ ± 1.39	46.98^c^ ± 1.05	2.9	3.6	21^***^	16^***^	0.27	6.39^**^	0.10	8.67^***^	47.53
	ARA	%CWR	3.30^a^ ± 0.16	3.64^b^ ± 0.12	3.61^b^ ± 0.12	3.89^c^ ± 0.1	9.4	6.9	39.37^***^	34.54^***^	0.41	2.88^***^	0.36	3.36^***^	19.06
	GAL	%CWR	0.67^a^ ± 0.14	0.85^b^ ± 0.14	0.93^b^ ± 0.11	1.10^c^ ± 0.11	38.8	29.4	20.15^***^	42.37^***^	0.01	4.98^***^	0.03	6.89^***^	25.54
	XYL	%CWR	19.94^b^ ± 0.71	19.38^a^ ± 0.65	19.42^ab^ ± 0.79	18.99^a^ ± 0.97	2.6	2.0	8.42^***^	8.48^***^	0.18	59.06^***^	0.35	2.91^***^	20.58

e *NDF, neutral detergent fiber; IVDMD, in vitro dry matter degradability; IVCWRD, in vitro cell wall residues degradability; ADL, acid detergent lignin; KL, Klason lignin; PCAest, esterified para-coumaric acid; FAeth, etherified ferulic acid; FAest, esterified ferulic acid; CL, cellulose; HC, hemicellulose; GLU, glucose; ARA, arabinose; GAL, galactose; XYL, xylose*.

f *When two samples show different letters after the mean, the difference between them is significant (normal letters, P < 0.05)*.

g *r^2^ value from the anova using the model (2)*.

* *significant at p < 0.05*,

** significant at p < 0.01 and

*** *significant at p < 0.001*.

Cultivation under the NI scenario significantly altered most traits, except for the structural sugar levels, relative to the I scenario, (Table 2). Plant height and biomass yield were much lower under the NI scenario. NDF increased while lignin contents in cell wall decreased, paralleled by an increase of both IVDMD and IVCWRD. The overall ß-O-4 yield decreased under the NI scenario, the S unit content decreased while the G unit content remained unchanged, leading to a decrease of the S/G ratio. In contrast, the H unit content increased, while the PCAest content decreased under the NI scenario. It is noteworthy that the scenario explained 49.45% of the observed variation for PCAest content (Table 2). Finally, under the NI scenario FAest and FAeth showed only a small reduction and the effect of the irrigation scenario explained only 8.0 and 6.6% of the observed variation in FAest and FAeth, respectively (Table 2).

It is worth noting that F271 and Cm484 responded differently to the irrigation scenarios (Table 2). The agronomical traits were more impacted by the irrigation scenarios in F271 (reduction of 16.6 and 36.6% of plant height and biomass yield, respectively, in the NI vs. I scenario) than in Cm484 (reduction of 8.3 and 15.7% of plant height and biomass yield, respectively, in NI vs. I scenario). The increase in IVDMD and IVCWRD was also more pronounced in F271 (increase of 8.9 and 26.7% of IVDMD and IVCWRD, respectively, in the NI vs. I scenario) than in Cm484 (increase of 1.1 and 18.4% of IVDMD and IVCWRD, respectively, in the NI vs. I scenario). In contrast, the increase in NDF under the NI scenario was more pronounced in Cm484 (9.7%) than in F271 (3.3%). Surprisingly, F271 and Cm484 showed similar responses to the irrigation scenarios for the lignin contents. These findings were in accordance to a low *r*^2^ (0.89 and 0.44% for KL.CWR and ADL.NDF, respectively), whereas a significant interaction between genotype and irrigation scenarios effect was observed.

### In the F271 × Cm484 RIL Progeny, Cell Wall Composition and Degradability Responded to the Irrigation Scenario

The agronomic and cell wall-related traits were then evaluated in the F271 × Cm484 RIL progeny (Table 3). The trait variation was higher in the RIL progeny than in the parental lines, illustrating the so-called transgression effect, except for IVDMD and ARA under the I condition. Moreover, a strong genotypic effect and medium to high heritabilities were observed for all traits. Importantly, large genotypic variability among the F271 × Cm484 RIL progeny was found in the general trend under both irrigation scenarios as shown by the minimal and maximal values (Table 3). The strongest heritabilities (h^2^ above 0.7) were observed for plant height, biomass yield, IVCWRD, KL.CWR, ADL.NDF, PCAest, FAest, CL.NDF, HC.NDF, and ARA. On the other hand, lowest heritabilities (h^2^ < 0.6) were observed for traits related to lignin structure, GLU, GAL, and XYL.

**Table 3:** Agronomical, cell wall composition, and degradability traits in the RIL progeny.

**Categories**	**Traits^a^**	**Units**	**Irrigated**			**Non-Irrigated**			**Genetic^b^**	**Irrigation scenario^b^**	**Genetic × Irrigation scenario^b^**	**Year^b^**	**Genetic × year^b^**	**Year × Irrigation scenario^b^**	**Residual^b^**	**h^**2c**^**
			**Mean**	**Min**	**Max**	**Mean**	**Min**	**Max**								
Agronomic	Plant height	cm	149.18 ± 18.59	99.16	197.97	127.07 ± 13.73	91.95	185.04	49.17^***^	29.04^***^	5.14^***^	0.44^***^	7.01^***^	0.08*	9.12	0.87
	Yield	t ha^−1^	3.01 ± 0.92	0.92	6.50	2.18 ± 0.6	0.99	4.76	46.02^***^	19.04^***^	7.46	0.19*	11.27^***^	0.00E+00	16.02	0.75
Cell Wall residue	NDF	%DM	53.14 ± 2.91	46.14	61.40	54.91 ± 2.52	48.40	60.74	45.48^***^	6.66^***^	15.05^***^	1.23^***^	8.79	0.43^**^	22.36	0.62
Degradability	IVDMD	%DM	49.44 ± 2.63	44.08	56.27	53 ± 2.56	45.42	59.40	27.11^***^	57.23^***^	3.85^*^	1.29^***^	6.65	1.75^***^	18.58	0.65
	IVCWRD	%CWR	33.42 ± 1.95	28.39	38.23	41.31 ± 2.07	35.88	46.54	31.42^***^	55.89^***^	2.86	1.68^***^	3.09	0.23^***^	7.91	0.77
Lignin content	KL.CWR	%CWR	15.44 ± 0.56	13.85	17.17	13.9 ± 0.59	12.48	15.31	36.91^***^	22.59^***^	12.22^***^	0.27^***^	2.87	0.84^***^	7.84	0.83
	ADL.NDF	%NDF	5.16 ± 0.4	4.07	6.43	4.12 ± 0.42	3.03	5.08	15.06^***^	68.05^***^	3.26^**^	0.08	2.82^*^	0.32^***^	6.61	0.88
Lignin structure	bO4	μmole g^−1^ KL	504.79 ± 63.72	313.91	652.76	482.36 ± 57.45	276.36	603.11	38.32^***^	2.66^***^	12.76	0.00E + 00	13.26	1.72^***^	31.28	0.56
	bO4.H	μmole g^−1^ KL	14.97 ± 1.65	9.86	21.24	18.06 ± 1.35	14.01	20.62	24.43^***^	34.03^***^	8.51	4.81^***^	6.33	1.19^***^	20.71	0.56
	bO4.G	μmole g^−1^ KL	248.24 ± 34.82	132.14	329.08	254.52 ± 30.07	152.30	309.80	38.2^***^	0.15	12.29	0.76^***^	12.86	2.87^***^	32.85	0.57
	bO4.S	μmole g^−1^ KL	241.94 ± 32.81	132.45	334.45	217.98 ± 30.2	115.28	287.12	36.04^***^	8.46^***^	12.57	2.12^***^	11.85	0.95^***^	28.01	0.54
	S/G		0.96 ± 0.06	0.81	1.14	0.85 ± 0.07	0.70	1.05	29.76^***^	29.28^***^	7.49	6.07^***^	6.91	0.31^**^	20.18	0.66
*p*-Hydroxycinnamic acids	PCAest	mg g^−1^ CWR	12.37 ± 1.09	9.51	14.91	9.39 ± 1.13	6.64	12.42	26.43^***^	55.61^***^	4.48^*^	0.15^**^	3.2	0.63^***^	9.49	0.82
	FAest	mg g^−1^ CWR	3.3 ± 0.4	2.16	4.37	3.17 ± 0.39	2.14	4.08	49.31^***^	2.81 ^***^	10.92^**^	0.63^***^	8.11	7.45^***^	20.77	0.73
	Faeth	mg g^−1^ CWR	2.54 ± 0.07	2.29	2.75	2.43 ± 0.07	2.26	2.65	27.54^***^	24.96^***^	11.54^***^	5.45^***^	7.58	2.73^***^	20.21	0.53
Structural sugars	CL.NDF	%NDF	50.51 ± 1.36	47.38	54.38	49.87 ± 1.44	46.44	53.92	52.38^***^	2.55^***^	9.47	0.12	10.35^**^	3.26^***^	21.87	0.74
	GLU	%CWR	38.35 ± 1.02	35.16	42.51	37.19 ± 0.89	34.79	40.02	30.75^***^	17.5^***^	13.49^**^	0.59^**^	10.41	0.28^*^	31.18	0.51
	HC.NDF	%NDF	44.28 ± 1.68	39.78	48.15	46.02 ± 1.8	40.87	50.53	51.33^***^	13.43^***^	7.41	0.07	8.37^**^	1.67^***^	17.72	0.79
	ARA	%CWR	3.45 ± 0.13	2.97	3.77	3.73 ± 0.16	3.23	4.11	34.97^***^	38.19^***^	7.17^**^	0.05	5.53	0.15^*^	13.94	0.78
	GAL	%CWR	0.74 ± 0.1	0.38	1.01	0.96 ± 0.11	0.59	1.24	25.3^***^	37.23^***^	9.29^*^	1.72^***^	6.9	1.06^***^	18.51	0.58
	XYL	%CWR	19.18 ± 0.47	16.92	20.56	19.04 ± 0.38	17.85	19.89	18.79^***^	1.28^***^	8.32^*^	32.83^***^	7.04	14.79^***^	16.95	0.49

a *NDF, neutral detergent fiber; IVDMD, in vitro dry matter degradability; IVCWRD, in vitro cell wall residues degradability; ADL, acid detergent lignin; KL, Klason lignin; PCA, para-coumaric acid; FA, ferulic acid; CL, cellulose; HC, hemicellulose; GLU, glucose; ARA, arabinose; GAL, galactose; XYL, xylose*.

b *r^2^ value from the anova using the model (2)*.

* *significant at p < 0.05*,

** significant at p < 0.01 and

*** *significant at p < 0.001*.

c *Broad-sense heritability*.

**Figure 1:**
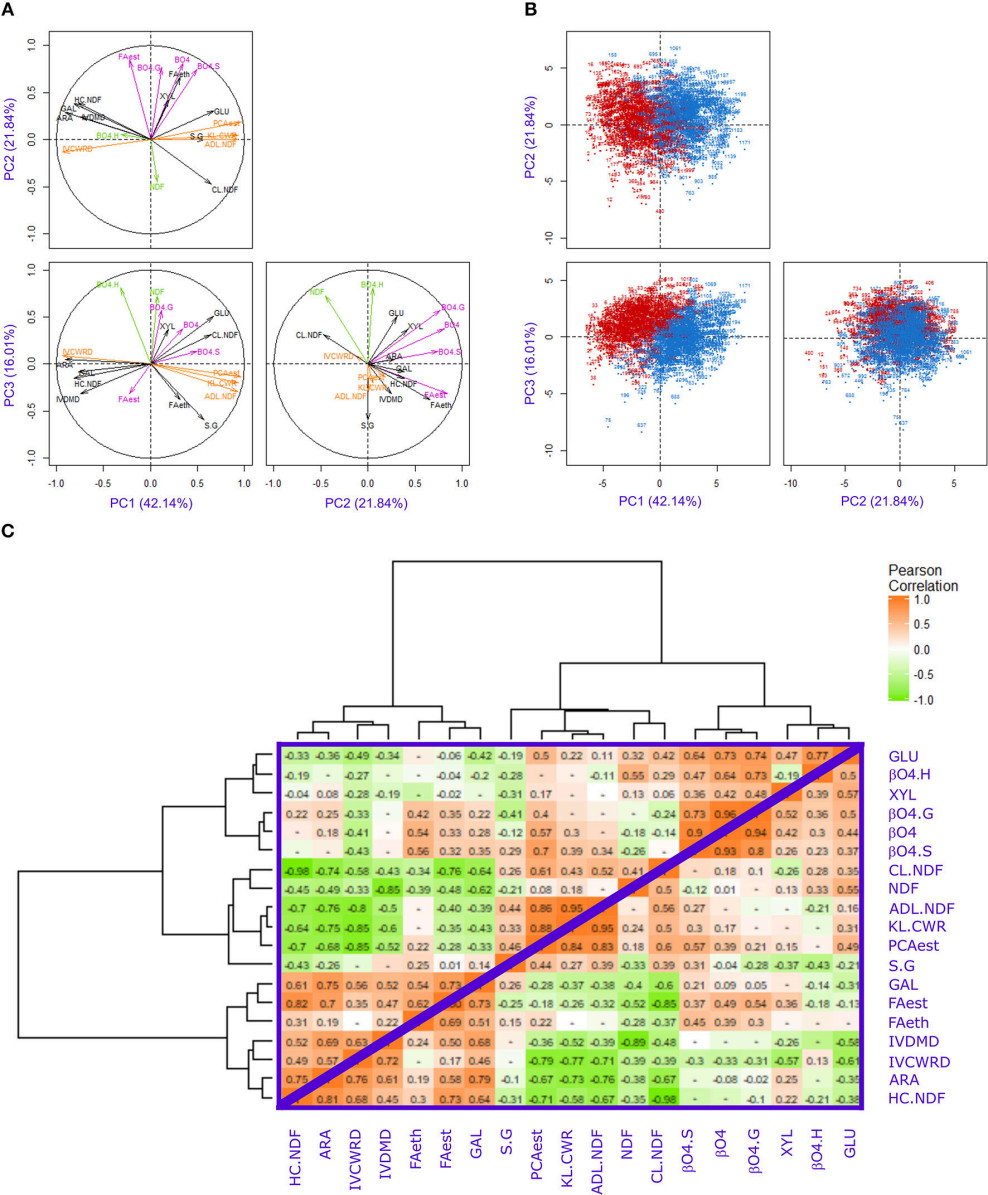
Principal Component Analysis (PCA) plots and correlation matrix of the investigated traits. **(A)** Distribution of the cell wall-related traits on the first, second, and third components, explaining 42.14, 21.84, and 16.01% of the variability observed, respectively. **(B)** Distribution of the F271 × Cm484 RIL progeny lines in Irrigated (blue) and Non-Irrigated (red) scenarios on the principal components PC1, PC2, and PC3. **(C)** Matrix of Pearson correlation in Irrigated (upper triangle) and Non-Irrigated (lower triangle) scenarios. The positive correlations were in orange and the negative in green, the color scale were from 0 to 1 or −1.

Principal component analysis (PCA) was performed with all the NIRS-estimated values for investigated traits under both irrigation scenarios (Figure 1) on the RIL progeny. The first three principal components (PCs) explained 80% of the variation of all the traits. The traits that contributed the most to the first principal component (PC1) were IVCWRD, KL.CWR, ADL.NDF, and PCAest (Figure 1A and Supplemental Table 1). These traits were strongly correlated (*r* ranging from 0.71 to 0.95 in both irrigation scenarios; Figure 1C) and the IVCWRD was negatively correlated to the KL.CWR, ADL.NDF, and PCAest. It is worth noting that these traits were strongly impacted by the irrigation scenarios (Table 3). Indeed, for these traits, the percentage of variance explained by the irrigation scenarios effect was always higher than that explained by the genotypic effect (Table 3). Hence, PC1 contributed to 59% of the irrigation scenario effect (Figure 1B).

The traits that contributed the most to the second principal component (PC2) were FAest, β-O-4 yield, β-O-4.G, and β-O-4.S (Figure 1A and Supplemental Table 1). The correlation between FAest and the other traits was lower (*r* ranging from 0.32 to 0.36) than that observed between the β-O-4 yield and the β-O-4.G and β-O-4.S contents (*r* ranging from 0.8 to 0.96 in the I scenario; Figure 1C). Furthermore, the percentage of variance explained by the irrigation scenarios was strikingly lower than that for the genotypic effect (Table 3). It should be noted that the irrigation scenario had no significant impact on β-O-4.G levels in both F271 × Cm484 RIL progeny and parental inbred lines (Table 2). Consistently, PC2 did not allow RILs cultivated under the I or the NI scenario to be distinguished (Figure 1B).

The traits that contributed most to the third principal component (PC3) were ß-O-4.H contents and NDF (Figure 1A and Supplemental Table 1). PC3 contributed to 16% of the irrigation scenarios effect and allowed a better separation of RILs cultivated under each irrigation scenario (Figure 1B). Overall, only a few traits (NDF, KL.CWR, FAest, FAeth, and GLU) showed strong (*r*^2^ > 10%) and significant effects for the interaction between genotype and irrigation scenarios (Table 3), suggesting that variation of the response to the irrigation scenario in the F271 × Cm484 RIL progeny occurred only for these traits.

### Fifteen Clusters Encompassed More Than Two Thirds of the 213 Constitutive QTLs Detected

**Figure 2:**
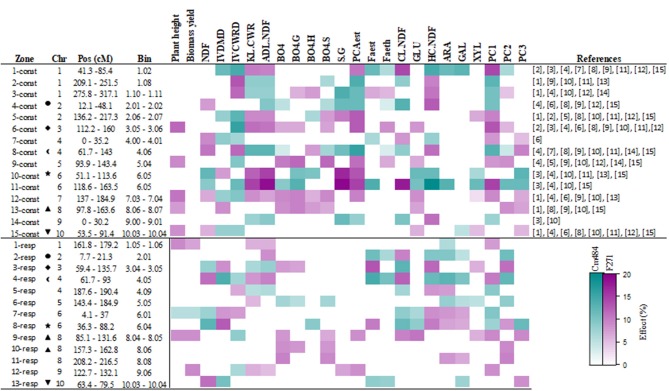
Summary of the clusters of constitutive and responsive QTLs identified for agronomic, cell wall composition, degradability, and PCA coordinate components traits. The color corresponds to the allele increasing the traits, F271 in magenta and Cm484 in cyan. The scale of colors represented the effect at the QTL position (Supplemental Table 2) over the variation of the corresponding trait on the RIL progeny lines (see materials and methods). The symbols correspond to co-localization between constitutive and responsive QTLs. The references correspond to: [1] Méchin et al., 2001; [2] Roussel et al., 2002; [3] Cardinal et al., 2003; [4] Fontaine et al., 2003; [5] Krakowsky et al., 2005; [6] Krakowsky et al., 2006; [7] Riboulet et al., 2008a; [8] Barrière et al., 2008; [9] Wei et al., 2009; [10] Truntzler et al., 2010; [11] Lorenzana et al., 2010; [12] Barrière et al., 2012; [13] Courtial et al., 2013; [14] Torres et al., 2015; [15] Leng et al., 2018.

Using a mixed linear model, we detected constitutive QTLs, when the “*m*_p_” term was significant [see equation (3) in Materials and Methods section]. Allelic variation present at a constitutive QTL for a given trait impacted the variation of this trait whatever the year and the irrigation scenario. For plant height, biomass yield and the 19 cell wall traits, a total of 213 constitutive QTLs were detected, spread over the 10 maize chromosomes (Supplemental Table 2 and Supplemental Figure 1). Among the 213 constitutive QTLs detected, 142 (i.e., two-thirds) mapped to 15 clusters (Figure 2 and Supplemental Table 2). These 15 clusters contained six to 14 QTLs for the 21 investigated traits. The 11-const cluster on chromosome 6 included 14 QTLs and showed the highest *r*^2^ among the clusters detected. At the agronomic level, 19 QTLs were detected for plant height, but only 5 co-localized with the 15 identified cluster. For biomass yield, 12 QTLs were detected, but none of them were localized within the 15 identified clusters. The 11 QTLs for IVCWRD, always co-localized with QTLs for KL.CWR, ADL.NDF, and PCAest, except in clusters 7-const, 9-const and 15-const (Figure 2 and Supplemental Table 2). Within cluster 7-const, the QTL for IVCWRD co-localized with QTLs for FAest, CL.NDF, and HC.NDF. At cluster 9-const, the QTL for IVCWRD co-localized with QTLs for β-O-4 yield, β-O-4G, β-O-4S, PCAest, FAeth, and GLU. It is worth noting that most of the constitutive QTLs co-localized with QTLs detected for every traits under either irrigated or non-irrigated scenarios (Supplemental Table 3), underlying the fact that overall, these constitutive QTLs are present whatever the irrigated scenario.

Interestingly, 12 out of the 13 QTLs for PC1 co-localized with the 15 identified clusters (Figure 2 and Supplemental Table 2) and coincided with QTLs detected for IVCWRD, KL.CWR, ADL.NDF, and PCAest. This was consistent with the fact that these traits were the major contributors to PC1 (Figure 1A, Supplemental Table 1). Seven QTLs for PC2 coincided with those for FAest, β-O-4, β-O-4.G, and β-O-4.S yield in the 15 identified clusters (Figure 2). This was consistent with the fact that these traits were the major contributors to PC2 (Figure 1A and Supplemental Table 1). Among the 11 identified QTLs for PC3, 7 co-localized with the 15 identified clusters (Figure 2). These QTLs coincided with those for ß-O-4.H and NDF, which were the traits that contributed the most to PC3 (Figure 1A and Supplemental Table 1). Hence, 26 QTLs for PC1, PC2, and PC3 coordinates summarized the 15 obtained clusters.

### Thirteen Clusters Encompassed More Than 60% of the 149 Responsive QTLs Detected

Using the same mixed linear model, we also detected responsive QTLs, when the “*m*_p_ x $e_{{\text{k}}^{\prime \prime }}$ term was significant [see equation (3) in Materials and Methods section]. Allelic variation at a responsive QTL explained the differences of response found for a given trait between plants carrying F271 alleles and those carrying Cm484 alleles depending on the environment. A total of 149 significant responsive QTLs were identified for all the individual traits (Supplemental Table 2 and Supplemental Figure 1). Among them, 93 (62%) clustered on 13 loci (Figure 2 and Supplemental Table 2). At the agronomic level, 6 responsive QTLs for plant height and 8 responsive QTLs for biomass yield were detected. However, only 3 responsive QTLs for plant height and 4 responsive QTLs for biomass yield co-localized with the 13 identified clusters. Interestingly, 5 of the 6 responsive QTLs for IVCWRD co-localized with responsive QTLs for other traits in the 13 clusters. The two clusters 7-resp and 9-resp included responsive QTLs for plant height, biomass yield, IVCWRD, structural sugars and PCAest. In contrast, the two clusters 4-resp and 5-resp grouped responsive QTLs for IVCWRD, lignin content, HC.NDF and CL.NDF. The cluster 4-resp also included responsive QTL for FAest and FAeth.

Responsive QTLs for PCs were also mapped (Supplemental Table 2 and Figure 2). Seven out of the eight responsive QTLs for PC1 co-localized in seven cluster (1-resp, 4-resp, 5-resp, 7-resp, 8-resp, 9-resp, and 12-resp) with responsive QTLs for traits that were the major contributors to PC1 (namely IVCWRD, KL.CWR, ADL.NDF, and PCAest; Figure 1A and Supplemental Table 1). Additionally, seven of the 11 responsive QTLs for PC2 co-localized with responsive QTLs for FAest (2-resp, 3-resp, 4-resp, and 8-resp), β-O-4, β-O-4.G, or β-O-4.S yield (3-resp, 6-resp, 10-resp, and 11-resp). Only three of the seven responsive QTLs for PC3 co-localized with clusters of responsive QTLs. At the 8-resp locus, a responsive QTL for PC3 co-localized with responsive QTLs for β-O-4.H and NDF which were the traits that contributed the most to PC3. However, at cluster 9-resp, the presence of a responsive QTL for PC3 was not associated with the presence of responsive QTLs for β-O-4.H nor for NDF.

It is worth noting that six of the 13 clusters of responsive QTLs did not co-locate with the 15 clusters of constitutive QTLs (Figure 2). Furthermore, the traits involved in “constitutive clusters” co-locating with “responsive clusters,” were not always the same. As such, the constitutive cluster 4-const, which encompassed 10 QTLs and the responsive cluster 2-resp which consisted of 8 QTLs, shared only 5 QTLs for common traits.

## Discussion

### Optimized and Accurate NIRS Equations Enable the Genetic Analysis of a Broad Number of Cell Wall-Related Traits in a Large Set of Maize Stover Samples

NIRS is routinely employed in a commercial setting for the assessment of complex forage quality traits in maize including the analysis of cell wall digestibility (reviewed in Torres et al., 2015). Several published examples highlight that cell wall-related traits can be accurately predicted in maize stover. This is the case for dry matter and cell wall degradability (Lübberstedt et al., 1997c; Riboulet et al., 2008b; Jung and Phillips, 2010), as well as for the traits quantified by the Van Soest chain (Dardenne et al., 1993; Lorenz et al., 2009; Jung and Phillips, 2010). The established equations in this study show also a high *r* of validation for the above-mentioned traits (*r* ranging from 0.77 to 0.95).

Lignin content has been estimated using both Van Soest (ADL.NDF) and Klason (KL.CWR) methods. As discussed in Zhang et al. (2011) and in accordance with Fukushima and Hatfield (2004) the KL procedure is more suitable for the global determination of lignin content, whereas the ADL procedure allows the proportion of the more condensed lignin fraction to be determined. Hames et al. (2003) (cited in Lorenz et al., 2009) reported NIRS predictive equations for lignin contents measured with both methods. In the present study, the *r* of validation or cross-validation for both lignin contents was high (*r* = 0.83). It is worth noting that Barrière et al. (2010) used NIRS equations to predict both lignin contents in a RIL progeny and detected only 2 common QTLs for both lignin contents among the 14 detected QTLs. In the present study, 85% of the QTLs for ADL.NDF and KL.CWR co-localized.

NIRS predictive equations for *p*-hydroxycinnamic acids contents have also been proposed (Riboulet et al., 2008b; Jung and Phillips, 2010; Lorenzana et al., 2010). All the proposed equations for PCAest content are satisfying with a *r* of validation ranging from 0.87 (Riboulet et al., 2008b) to 0.95 (Lorenzana et al., 2010). Our predictive equation is in the same order of magnitude (*r* of validation = 0.78). In contrast, predictive equations for FA contents are generally unreliable. Thus, the equation proposed by Lorenzana et al. (2010) using stover from the studied mapping population has a *r*^2^ of calibration of 0.04 for FA content. Jung and Phillips (2010) were able to report a *r*^2^ of 0.95 by using for calibration samples from different plant parts harvested at different maturity stages (from immature leaf blade to mature stem samples). This *r*^2^ of validation allowed FA content to be distinguished between organs but not the variation of FA content within the same organ harvested at the same stage. Riboulet et al. (2008b) proposed NIRS predictive equation for FA contents for maize stover harvested at silage stage with a *r*^2^ of calibration ranging from 0.64 to 0.66. In the present study, the predictive NIRS equations developed are robust, especially for FAest content (*r* of validation = 0.83, thus *r*^2^ = 0.69).

Thioacidolysis is a biochemical protocol to assess lignin structure (Lapierre et al., 1986). In this study, we proposed for the first time NIRS predictive equations for monolignol composition based on the thioacidolysis analysis of maize stover. β-O-4 yield and β-O-4 and monolignol content are robustly estimated with our established equations (*r* of validation ranging from 0.71 to 0.90). The prediction of the S/G ratio is slightly less robust but is nevertheless above 0.6 (*r* = 0.66).

Overall, the NIRS predictive equations proposed herein are novel, robust and accurate to predict cell wall-related trait on maize stover harvested at silage stage. They are dedicated to maize stover samples harvested at silage stage and were developed in parallel with those recently presented in El Hage et al. (2018) dedicated to maize internodes harvested at silage stage.

### The NI Scenario Has a Strong Impact on Agronomic and Cell Wall Related Traits but Does Not Affect Their Correlation Structure

The irrigation scenario significantly affected both agronomic and cell wall-related traits. Indeed, average biomass yield and plant height were reduced up to 27.5 and 14.8%, respectively. In sorghum, Perrier et al. (2017) noticed a reduction of plant height ranging from 17.8 to 23.4% when plants were submitted to a similar NI scenario. Furthermore, we observed a significant reduction of lignin and the PCAest contents under the NI relative to I conditions. This is consistent with previous observations on maize (Emerson et al., 2014; El Hage et al., 2018), sorghum (Perrier et al., 2017), sugarcane (dos Santos et al., 2015), and miscanthus (Emerson et al., 2014; van der Weijde et al., 2017).

Although the irrigation scenarios were contrasted enough to provoke significant agronomic and cell wall modifications, the overall structure of the correlations between the investigated cell wall related traits was not affected by the NI scenario (Figure 1C). Whatever the irrigation scenario, lignin, and PCAest contents were tightly correlated as previously described (Hatfield et al., 1999). These two traits were strongly and negatively correlated to cell wall degradability. Zhang et al. (2011) and El Hage et al. (2018) have suggested that lignin and PCAest contents may have distinct roles on cell wall degradability. It is noteworthy that FA contents were less correlated to cell wall degradability, despite their established role in cell wall cross linking (Hatfield et al., 2017) which was reported to be critical for cell wall degradability (Grabber et al., 1998; Jung and Casler, 2006).

The impact of β-O-4 yield on cell wall degradability is still subject to debate and has been reported by Besombes and Mazeau (2005) and Zhang et al. (2011) as limiting. In the present study, the β-O-4 yield was mildly correlated with cell wall degradability whatever the irrigation scenario. We observed that the NI scenario had no impact on β-O-4.G and β-O-4.S subunits, leading to the same S/G ratio in both irrigation conditions. The observed modifications on lignin and PCAest contents under the NI scenario led to a similar expected amount of S units in lignin acylated by PCAest (16.5 and 14.7% in the I and NI scenarios, respectively). The amount of H units in lignin, however, was increased by the NI scenario. This is a signal observed when plants are submitted to different sources of stress (reviewed in Cabane et al., 2012). For instance, H subunits increased when poplar plants were under ozone treatment (Cabané et al., 2004). H subunits are also terminal units of lignin polymer and their increase could contribute to the parceling out of the lignin polymer. In addition, the fact that the FAest content was not impacted under the NI scenario, while the lignin content was reduced, could reflect a lignin more fragmented under stress. Mottiar et al. (2016) suggested that shorter lignin chains should be more prone to degradability. Hence, the increase in cell wall degradability under the NI scenario very likely reflects a decrease in lignin and PCAest contents but might also be due to modifications of the lignin structure, which appeared to be more fragmented under the NI scenario. Indeed, etherified ferulic acids represent the ferulic primers used for lignin anchoring. Thus, if the number of ferulic bonds is comparable under both environments while the lignin content is higher in the irrigated one (Table 3), then the lignin chains on each primer must be longer to explain the higher lignin content.

### A Complex Genetic Architecture of Cell Wall Composition and Degradability Traits Over the Irrigation Scenarios and Their Responses to the NI Scenario

Thus far, numerous QTL studies have been reported on traits related to cell wall composition and degradability for maize at silage stage (Lübberstedt et al., 1997a,c; Bohn et al., 2000; Barrière et al., 2001, 2008, 2012; Méchin et al., 2001; Roussel et al., 2002; Cardinal et al., 2003; Fontaine et al., 2003; Papst et al., 2004; Cardinal and Lee, 2005; Krakowsky et al., 2005, 2006; Riboulet et al., 2008a; Wei et al., 2009; Lorenzana et al., 2010; Courtial et al., 2014; Torres et al., 2015; Leng et al., 2018). These studies allowed to map over 400 QTLs all over the maize genome (Barrière et al., 2008). The present study allows the identification of 15 clusters of constitutive QTLs over the years and the irrigation scenarios for traits related to cell wall composition and degradability. All the loci detected were already mentioned in previous publications (Figure 2). Thus, in bin 3.05 (cluster 6-const), 60% of the previous studies mapped a QTL for cell wall degradability or composition. Torres et al. (2015) already mentioned that this genomic region was often identified for these types of traits. Using the F271 × Cm484 RIL progeny, the strongest QTL region was localized on bin 6.05 (cluster 11-const). This region has already been identified as a hotspot in several studies (Roussel et al., 2002; Courtial et al., 2013, 2014) using the F271 × F288 RIL progeny. This suggests that the alleles from the parental inbred line F271 used shared by both RIL populations, confers some common cell wall properties. Furthermore, the parental line F271 was found to be less degradable than the parental line Cm484, in agreement with previous studies (Méchin et al., 2000; El Hage et al., 2018). However, it is worth noting that the constitutive QTL alleles conferring a higher cell wall degradability did not always come from the better degradable inbred line Cm484. Thus, the allele from F271 conferred an increase of IVCWRD for three of the 15 detected clusters of constitutive QTLs (2-const, 3-const, and 8-const).

Several biomass yield QTLs were detected, but none of them mapped among the constitutive clusters for biomass quality detected in the present study (Supplemental Figure 1 and Supplemental Table 2). This result suggests that cell wall degradability and its response to water deficit could be selected without impacting biomass yield (Baldy et al., 2017; van der Weijde et al., 2017). Additionally, numerous constitutive QTLs for IVCWRD, LK.CWR and/or, ADL.NDF co-localized in a manner that is consistent with the strong correlation observed between the cell wall degradability and the lignin content. In contrast, QTLs for IVCWRD were not always co-localized with QTLs for lignin content in agreement with previous observations (Truntzler et al., 2010; Penning et al., 2014). We noticed that QTLs for IVCWRD and PCAest more often co-localized. For instance, the cluster 9-const encompassed QTLs for IVCWRD, β-O-4 yield, PCAest, and FAest, suggesting an independent potential role of these traits in the variation of the cell wall degradability as previously described (Grabber, 2005; Zhang et al., 2011). Collectively, our results highlight the complexity of the genetic determinism of cell wall related traits (Barrière et al., 2008; Torres et al., 2015).

In maize, QTLs for drought tolerance of grain yield have been largely studied and reported (Ribaut and Ragot, 2007; Collins et al., 2008; Ribaut et al., 2010; Millet et al., 2016). The present study identifies for the first time QTLs for traits related to cell wall degradability and composition in response to water deficit. It is noteworthy that while the interaction “genotype x irrigation scenario” among the RIL progeny only marginally contributed to the observed trait variation, the use of a mixed linear model (Alimi et al., 2013) allowed the detection of significant responsive QTLs for all traits. Interestingly, only half of the responsive QTL clusters co-localized with constitutive QTL clusters, suggesting that the genetic determinism and the molecular mechanisms involved in cell wall development are different from those involved in responses to water deficit. Furthermore, our results show that the lignin content does not explain all the variation of the cell wall degradability in response to the irrigation scenario. Some responsive QTLs for IVCWRD co-localized with responsive QTLs for biomass yield, and in each case, alleles that increased IVCWRD in response to the NI scenario decreased the biomass production. These observations will be very helpful for the selection of high yielding drought tolerant lines with improved cell digestibility.

## Author Contributions

MR, VM, and SC designed the study and supervised data collection. FE, M-PJ, AB, SL, CH, SC, VM, and MR collected the samples. CB and CP constructed the maize RIL population. YG, M-PJ, EG, and VC carried out the biochemical trait quantification and the genotyping. LV, FE, MF, LM, and MR conducted the genetic study. LV, SC, VM, and MR wrote the manuscript.

### Conflict of Interest Statement

The authors declare that the research was conducted in the absence of any commercial or financial relationships that could be construed as a potential conflict of interest.

## Supplementary Material

Supplemental Table S1 Principal component analysis.

Supplemental Table S2 Constitutive and responsive QTLs details.

Supplemental Table S3 QTL detection per irrigation scenario.

Supplemental Figure S1 Representation of the constitutive and responsive QTLs detected for the agronomical and cell-wall related traits.
